# Modified Shock Index as an Indicator for Prognosis Among Sepsis Patients With and Without Comorbidities Presenting to the Emergency Department

**DOI:** 10.7759/cureus.20283

**Published:** 2021-12-08

**Authors:** KJ Devendra Prasad, Thamminaina Abhinov, KC Himabindu, K Rajesh, DGSR Krishna Moorthy

**Affiliations:** 1 Department of Emergency Medicine, Sri Devaraj Urs Medical College, Kolar, IND

**Keywords:** comorbidities, critical care, sepsis, respiration, shock

## Abstract

Objectives: Modified shock index (MSI) is a simple bedside tool used in the emergency department. There are a few studies suggesting MSI as a good prognostic indicator than shock index in sepsis patients. However, there is not enough research emphasizing the role of MSI in patients with comorbidities. Hence, this study aims to assess the predictive validity of MSI in predicting the prognosis of sepsis patients with and without co-morbidities.

Methods: From January to December 2020, a prospective observational study was conducted in a tertiary care teaching hospital. Patients with sepsis diagnosed based on systemic inflammatory response syndrome criteria and quick sequential organ failure assessment (qSOFA) were included. The need for mechanical ventilation and step down from the intensive care unit were outcome variables, MSI was considered as a predictor variable, and co-morbidities as an explanatory variable.

Results: Among people with co-morbidities, the MSI value on arrival to the emergency department had fair predictive validity in predicting the need for mechanical ventilation after 24 hours, as indicated by the area under the curve of 0.749 (95% CI: 0.600-0.897; p-value = 0.002) and a sensitivity of 68.75% in predicting mechanical ventilation after 24 hours (MSI ≥ 1.59). Among people without co-morbidities, the MSI value on arrival to the emergency department had fair predictive validity in predicting the need for mechanical ventilation after 24 hours, as indicated by the area under the curve of 0.879 (95% CI: 0.770-0.988; p-value <0.001) and a sensitivity of 83.33% in predicting the need for mechanical ventilation after 24 hours (MSI ≥ 1.67).

Conclusion: MSI can be used as an indicator in predicting the prognosis of sepsis patients in the emergency department. A simple bedside calculation of the MSI can indicate the need for mechanical ventilation and step down from the intensive care unit after 24 hours in patients with co-morbidities and without co-morbidities.

## Introduction

Sepsis is a life-threatening condition that can lead to multi-organ dysfunction initiated by the dysregulated response of the host to infection, with potent forms such as severe sepsis and septic shock [1]. Sepsis and septic shock are medical emergencies that require immediate recognition and management [2]. Based on the Surviving Sepsis Campaign 2021, “the first hour for the identification and starting the management of sepsis starts from the patient’s arrival at triage” [3]. The shock index (SI) is an easy bedside tool that is calculated by “dividing heart rate (HR) over systolic blood pressure (SBP),” and the modified shock index (MSI) is calculated by “dividing heart rate over mean arterial pressure (MAP).” MAP is the recommended indicator to be followed for deciding fluid resuscitations and vasopressors titration as it is considered a better marker for organ perfusion than SBP or diastolic blood pressure (DBP) alone [4]. A high MSI value indicates stroke volume and low systemic vascular resistance (SVR), which reflects hyperdynamic circulation. This might indicate that the patient is in compensating phase, and the decompensation can occur rapidly. A low value of MSI indicates that the SI and SVR are high, which indicates the patient is in a hyperdynamic state; this can be a sign of severe sepsis [5].

MSI considers valuable information related to cardiovascular and hemodynamic stability by integrating HR, SBP, and DBP, which makes it an inclusive tool for assessment [6]. A study by Torabi et al. showed that in cases of emergency severity index level 3 patients, age, SI and SBP were better in predicting mortality compared to SI or MSI [7]. A study by Jayaprakash et al. showed that elevated MSI value in patients with early sepsis was associated with the occurrence of myocardial dysfunction and mortality [8].

Despite all the advances, there is no consensus on when and where MSI has a role in the emergency department. Despite the established association between sepsis and pre-existing comorbid conditions, limited information is available about the survival impact on sepsis patients. Most retrospective studies are done for prognosticating patients presenting to the emergency department. There is a shortage of prospective studies from India. The current study is an attempt to study the role of MSI in sepsis patients with and without co-morbidities.

## Materials and methods

Study design and setting

A prospective observational study was conducted in the department of emergency medicine. The data collection for this study was conducted between January 2020 and December 2020.

Ethics statement

This study was approved by the institutional human ethics committee and institutional review board of Sri Devaraj Urs Medical College (reference number: SDUMC/KLR/IEC/277/2019-20). Data confidentiality was maintained and written informed consent was obtained from the patients.

Study population

The study participants were patients presenting with features of sepsis to the emergency department.

Sample size

The sample size was calculated assuming the expected mortality of sepsis patients as 19.8% as per Jayaprakash et al.'s study [8]. The predictive validity was assessed by area under the curve (AUC) value of 0.75 against a null value of 0.5, 95% power, and 5% two-sided alpha error. As per the above-mentioned calculation, the required sample was 107. To account for a loss to follow up of 10%, another 11 subjects were included. To perform subgroup analysis based on the presence or absence of co-morbidities, we had not less than 118 subjects each with and without co-morbidities.

Study protocol

Participants were patients diagnosed with sepsis as per systemic inflammatory response syndrome (SIRS) criteria and quick sequential organ failure assessment (qSOFA) score and were in the age group of 18 years and above. Pregnant women, patients on immunosuppressive drugs, and patients with a history of trauma were excluded from the study. Baseline investigations like complete blood count and physical examination were done. SIRS was considered when fulfilling at least two of the following four criteria: “fever >38.0°C or hypothermia <36.0°C, tachycardia >90 beats/minute, tachypnea >20 breaths/minute, and leucocytosis >12*109/L or leucopenia <4*109/L.” The qSOFA score is a bedside tool that can identify patients with suspected sepsis who are at higher risk of developing a poor outcome outside the intensive care unit (ICU). It has three criteria, with one point for each: “low blood pressure (SBP ≤ 100 mmHg), increased respiratory rate (≥22 breaths per min), or altered mental status (Glasgow Coma Scale < 15).” MSI is calculated by “dividing heart rate (HR) over mean arterial pressure (MAP).” Patients with sepsis are identified mainly based on SIRS criteria. qSOFA is taken into consideration to parallelly prognosticate the patient. Patients stepping down from ICU and the need for mechanical ventilation were considered proxy variables for the severity of sepsis. Patients with severe sepsis will require mechanical ventilation and a longer duration of ICU care. Hence, these were considered as a proxy for sepsis severity.

Measurement of key outcome variables and data analysis

The need for mechanical ventilation after 24 hours and the patient stepping down from ICU after 24 hours were considered as primary outcome variables. The MSI value on arrival to the emergency department was regarded as the secondary outcome variable. Co-morbidities were considered as the primary explanatory variable. The association between explanatory variables and categorical outcomes was assessed by cross-tabulation and comparison of percentages. The MSI value on arrival to the emergency department in predicting the need for mechanical ventilation after 24 hours and patient stepdown from ICU after 24 hours was assessed by receiver operative curve (ROC) analysis. The AUC with its 95% CI and the p-value is presented. Based on the ROC analysis, it was decided to consider 1.67, 1.59, 1.35, and 1.29 as the cut-off values. The sensitivity, specificity, and diagnostic accuracy of the screening test with the fixed cut-off values along with their 95% CI were presented. P-value <0.05 was considered statistically significant. coGuide version 1.0 (coGuide, Bengaluru, Karnataka) was used for statistical analysis [9].

## Results

The final analysis included 235 subjects. Among the study population, the mean age was 56.12 ± 17.28 years. Of the participants, 139 (59.15%) were male and the remaining 96 (40.85%) were female.

Among the study participants, the majority (53.52%) of participants reported type 2 diabetes mellitus as co-morbidity, followed by chronic kidney disease as 10.21% (Table 1).

**Table 1:** Summary of chief complaints in the study population (n = 235). ICU - intensive care unit; HDU - high dependency unit; qSOFA - quick sequential organ failure assessment.

Parameter	Summary, n (%)
Chronic kidney disease	24 (10.21%)
Chronic liver disease	4 (1.7%)
Congestive cardiac failure	3 (1.28%)
Type 1 diabetes mellitus	1 (0.43%)
Type 2 diabetes mellitus	38 (53.52%)
Mechanical ventilation after 24 hours	28 (11.97%)
Mechanical ventilation after 72 hours	13 (5.53%)
Mechanical ventilation in the emergency department	26 (11.06%)
Patient step down from ICU/HDU units after 24 hours	114 (48.51%)
Step down at 72 hours	114 (48.51%)
Modified shock index at the time of disposition from the emergency department (mean ± SD)	1.25 ± 0.33
Modified shock index value on arrival to the emergency department (mean ± SD)	1.47 ± 1.11
qSOFA score (mean ± SD)	1.56 ± 0.57

**Figure 1:**
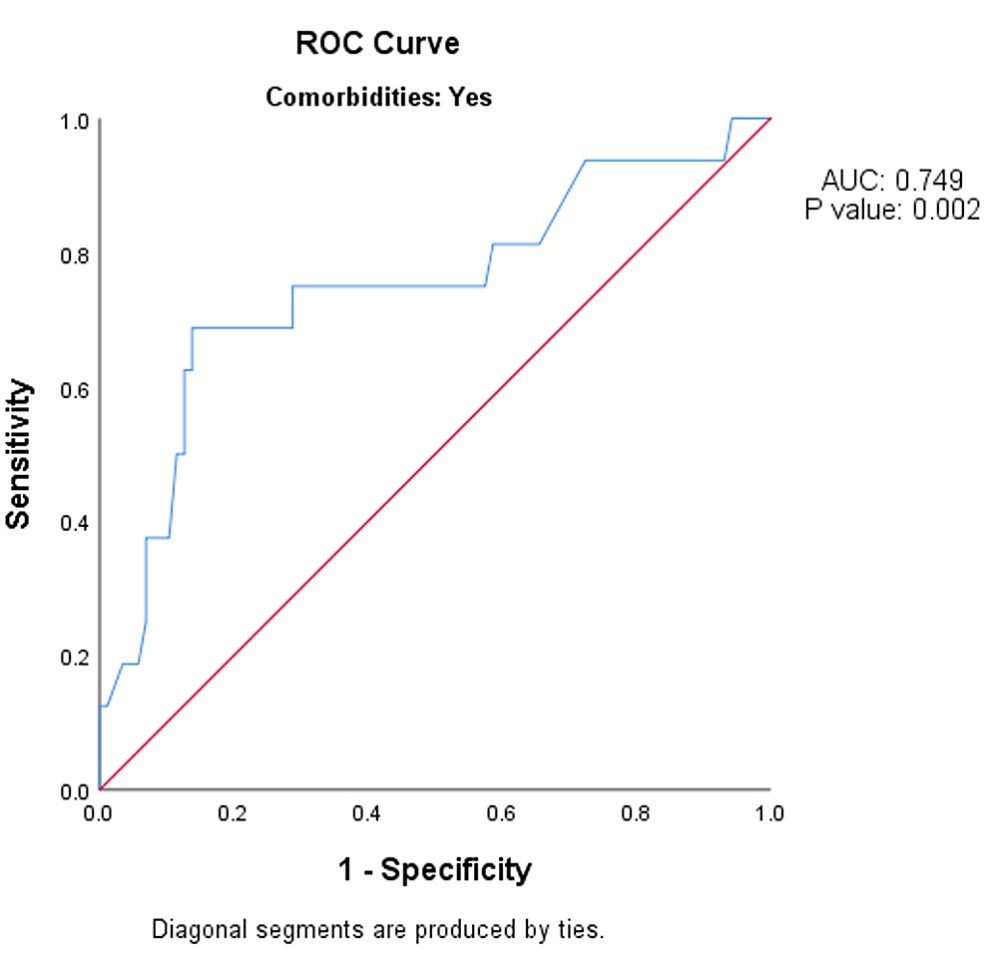
ROC analysis of predictive validity of modified shock index value on arrival to the emergency department in predicting the need for mechanical ventilation after 24 hours among people with co-morbidities (n = 103). ROC - receiver operative curve; AUC - area under the curve.

Among people with co-morbidities, the MSI value on arrival to the emergency department had the same (fair) predictive validity in predicting the need for mechanical ventilation after 24 hours, as indicated by the AUC of 0.749 (95% CI: 0.600-0.897; p-value = 0.002) (Figure 1).

**Figure 2:**
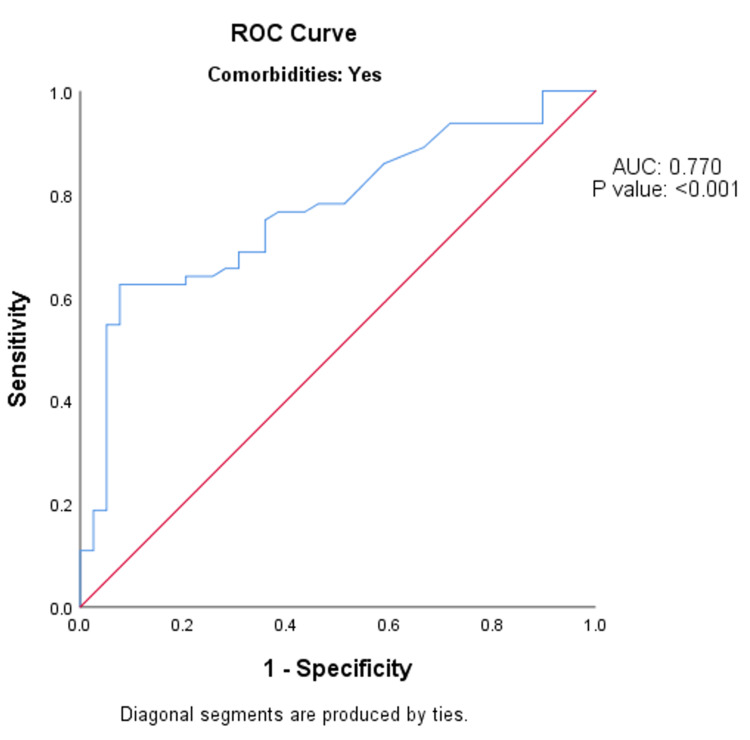
ROC analysis of predictive validity of modified shock index value on arrival to the emergency department in predicting the patient stepdown from ICU after 24 hours among the people with co-morbidities (n = 103). ROC - receiver operative curve; AUC - area under the curve.

Among the people with co-morbidities, the MSI value on arrival to the emergency department had the same (fair) predictive validity in predicting the patient stepdown from ICU after 24 hours, as specified by the AUC of 0.770 (95% CI: 0.678-0.862; p-value <0.001) (Figure 2).

Among the people with co-morbidities, the MSI value on arrival to the emergency department of 1.59 and above had a sensitivity of 68.75% in predicting mechanical ventilation after 24 hours. Specificity was 86.21%, the false-positive rate was 13.79%, the false-negative rate was 31.25%, the positive predictive value was 47.83%, the negative predictive value was 93.75%, and the total diagnostic accuracy was 83.50%. The MSI value on arrival to the emergency room of less than or equal to 1.35 had a sensitivity of 82.05% in predicting patient stepdown from ICU after 24 hours. Specificity was 51.56%, the false-positive rate was 48.44%, the false-negative rate was 17.95%, the positive predictive value was 50.79%, the negative predictive value was 82.50%, and the total diagnostic accuracy was 63.11% (Table 2).

**Table 2:** Predictive validity of modified shock index value on arrival to the emergency department in predicting outcomes among the people with and without co-morbidities (n = 235).

Parameter	With co-morbidities (N = 103)		Without co-morbidities (N = 132)	
	Need for mechanical ventilation after 24 hours (95% CI) (≥1.59)	Patient stepdown from ICU after 24 hours (95% CI) (≤1.35)	Need for mechanical ventilation after 24 hours (95% CI) (≥1.67)	Patient stepdown from ICU after 24 hours (95% CI) (≤1.29)
Sensitivity	68.75% (41.34% to 88.98%)	82.05% (66.47% to 92.46%)	83.33% (51.59% to 97.91%)	74.67% (63.30% to 84.01%)
Specificity	86.21% (77.15% to 92.66%)	51.56% (38.73% to 64.25%)	81.51% (73.36% to 88.04%)	73.68% (60.34% to 84.46%)
False-positive rate	13.79% (7.34% to 22.85%)	48.44% (35.75% to 61.27%)	18.49% (11.96% to 26.64%)	26.32% (15.54% to 39.66%)
False-negative rate	31.25% (11.02% to 58.66%)	17.95% (7.54% to 33.53%)	16.67% (2.09% to 48.41%)	25.33% (15.99% to 36.70%)
Positive predictive value	47.83% (26.82% to 69.41%)	50.79% (37.89% to 63.62%)	31.25% (16.12% to 50.01%)	78.87% (67.56% to 87.67%)
Negative predictive value	93.75% (86.01% to 97.94%)	82.50% (67.22% to 92.66%)	97.98% (92.89% to 99.75%)	68.85% (55.71% to 80.10%)
Diagnostic accuracy	83.50% (74.89% to 90.08%)	63.11% (53.03% to 72.41%)	81.68% (73.98% to 87.89%)	74.24% (65.91% to 81.46%)

**Figure 3:**
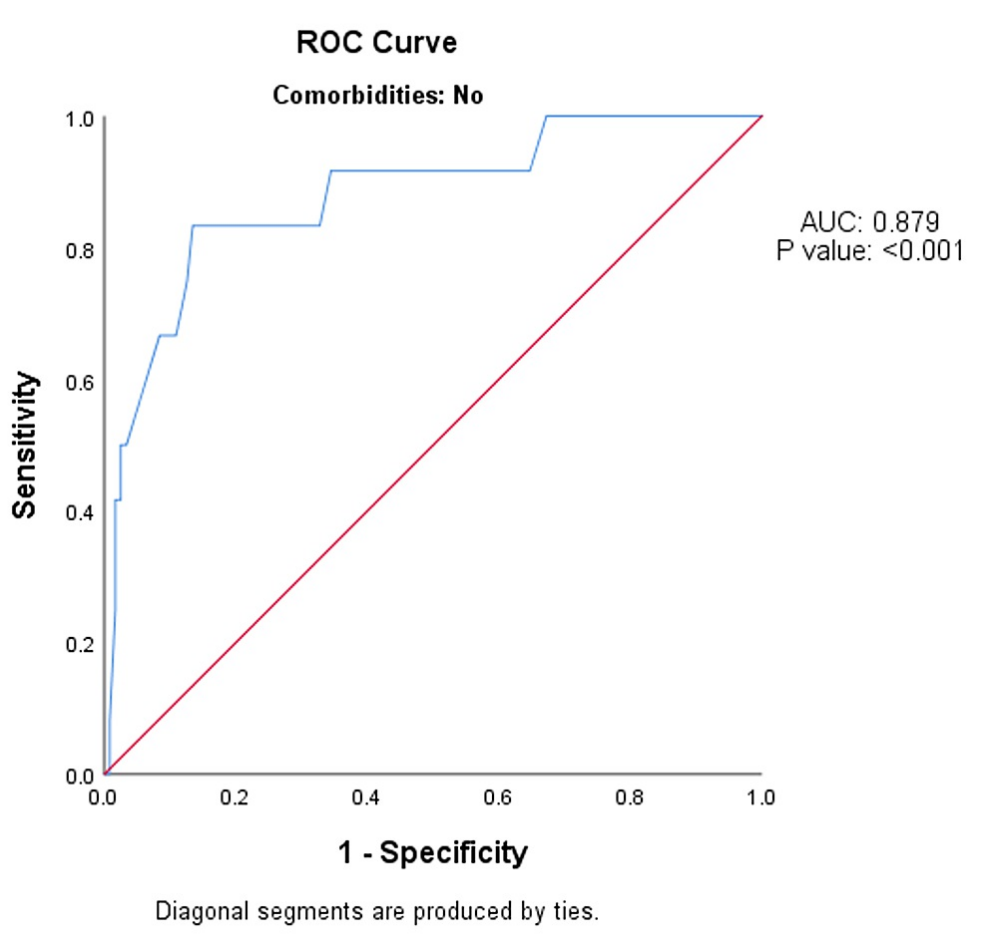
ROC analysis of predictive validity of modified shock index value on arrival to the emergency department in predicting the need for mechanical ventilation after 24 hours among people without co-morbidities (n = 131). ROC - receiver operative curve; AUC - area under the curve.

Among people without co-morbidities, the MSI value on arrival to the emergency department had the same (fair) predictive validity in predicting the need for mechanical ventilation after 24 hours, as indicated by the AUC of 0.879 (95% CI: 0.770-0.988; p-value <0.001) (Figure 3).

**Figure 4:**
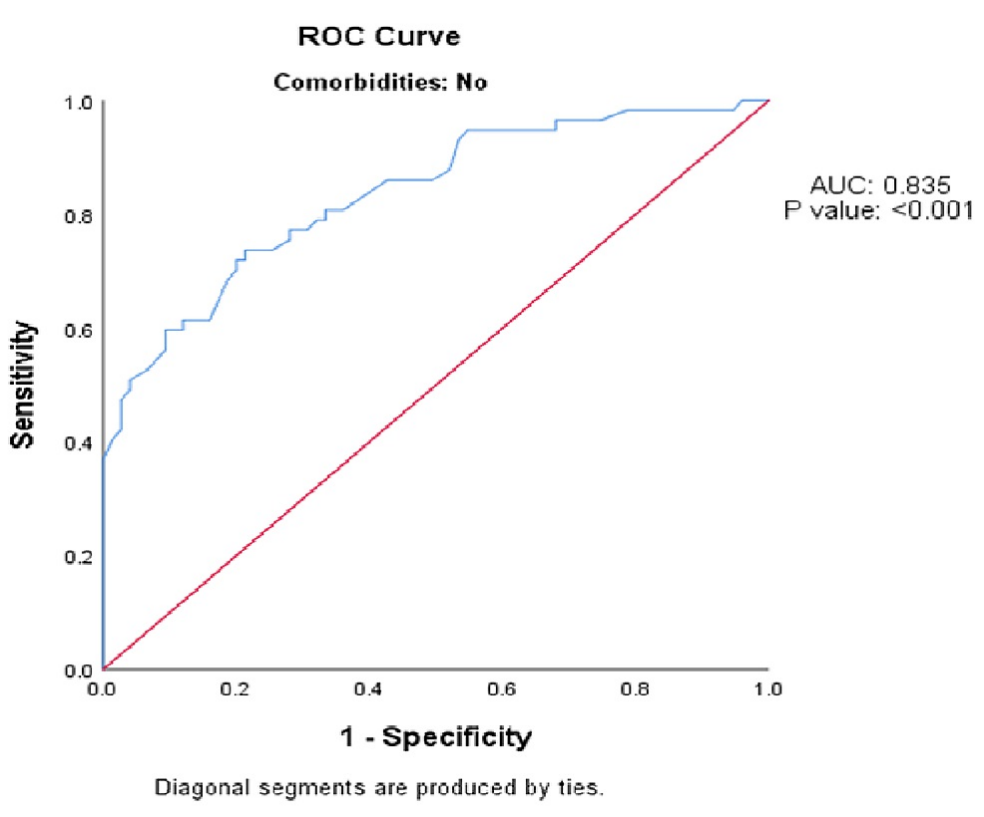
ROC analysis of predictive validity of modified shock index value on arrival to the emergency department in predicting the patient stepdown from ICU after 24 hours among the people without co-morbidities (n = 132). ROC - receiver operative curve; AUC - area under the curve.

Among people without co-morbidities, the MSI value on arrival to the emergency department had the same (fair) predictive validity in predicting the patient stepdown from ICU after 24 hours, as indicated by the AUC of 0.835 (95% CI: 0.790-0.988; p-value <0.001) (Figure 4).

Among the people without co-morbidities, the MSI value on arrival to the emergency department of 1.67 and above had a sensitivity of 83.33% in predicting the need for mechanical ventilation after 24 hours. Specificity was 81.51%, the false-positive rate was 18.49%, the false-negative rate was 16.67%, the positive predictive value was 31.25%, the negative predictive value was 97.98%, and the total diagnostic accuracy was 81.68%. The MSI value on arrival to the emergency room of less than or equal to 1.29 had a sensitivity of 74.67% in predicting patient stepdown from ICU after 24 hours. Specificity was 73.68%, the false-positive rate was 26.32%, the false-negative rate was 25.33%, the positive predictive value was 78.87%, the negative predictive value was 68.85%, and the total diagnostic accuracy was 74.24% (Table 2).

## Discussion

Despite recent advances in emergency care, existing literature suggests that sepsis still remains a substantial burden around the globe [10]. Every year, sepsis causes more than 6 million deaths worldwide, and it is one of the most costly diseases treated in the hospital [11]. Sepsis excessively affects elderly patients, those with severe comorbidities, and those patients who have impairment in functional status [12]. The shock index is used in hypovolemic shock patients at the early stages. When systolic blood pressure is used in shock index, another critical factor, DBP, may be neglected. Clinically, mean blood pressure can best represent tissue perfusion status.

This study was conducted to determine the role of MSI in predicting the need for mechanical ventilation and patient step down from ICU after 24 hours among patients with and without co-morbidities. This study showed that among people with co-morbidities, the MSI value on arrival to the emergency room had good predictive validity in predicting the need for mechanical ventilation after 24 hours. Predictive validity for patients stepping down from ICU after 24 hours was good. Similar findings were seen in the study by Althunayyan et al. [4]. The researchers did a retrospective cohort study among 274 febrile patients attending the ER. The researchers concluded that MSI acted as a good predictor of triaging of febrile patients. Liu et al. found that MSI performed better than either SI or heart rate and blood pressure alone in predicting mortality in emergency patients [5]. Singh et al., in their prospective study, found that MSI scores below 0.7 and above 1.3 were associated with a significantly increased mortality rate [6]. Available literature shows that 54% to 65% of all sepsis patients have comorbidities and these impact their clinical outcomes [13]. Some studies have found that comorbid conditions like cancer, HIV, diabetes, and alcohol usage may impact disease acceleration in sepsis. It was found that co-morbidities influence the risk and outcome of sepsis [14] and that cumulative co-morbidities are associated with more significant organ dysfunction [15,16]. In a retrospective cohort study of critically ill surgical patients with sepsis, Pittet et al. found that similar to acute illness severity, the type and number of co-morbidities were independently associated with mortality [17]. Among 17% of sepsis patients in the United States, Canada, and Europe, cancer has been documented as a comorbid condition [13]. The strength of this current study was patients with and without co-morbidities had been included, and subgroup analysis was done, which showed that irrespective of the associated co-morbidities, MSI acts as a good predictor of prognosis of sepsis patients.

Limitations

The limitation of the current study is that it was a single-center study on a limited sample. The etiology of the sepsis has not been described, which might influence the outcome variables. A multicenter study involving a huge sample size to determine the predictive validity of MSI is recommended. In the future, studies with a vast sample size covering the various clinical spectrum of presentation are advised to study the role of MSI.

## Conclusions

In conclusion, the MSI, a simple index that can be calculated at the bedside, acts as a fair predictor of the prognosis of patients with sepsis irrespective of the co-morbidity status. Hence, MSI can be utilized in the emergency department for the management of sepsis patients.
